# Trends in child growth failure among children under five years of age in Ethiopia: Evidence from the 2000 to 2016 Demographic and Health Surveys

**DOI:** 10.1371/journal.pone.0254768

**Published:** 2021-08-05

**Authors:** Tolesa Bekele, Patrick Rawstorne, Bayzidur Rahman

**Affiliations:** 1 School of Population Health, University of New South Wales, Sydney, Australia; 2 Department of Public Health, College of Medicine and Health Sciences, Ambo University, Oromia, Ethiopia; 3 Kirby Institute, University of New South Wales, Sydney, Australia; University of Mississippi Medical Center, UNITED STATES

## Abstract

**Introduction:**

In a majority of low- and middle-income countries (LMICs), levels of child growth failure (CGF) have steadily declined since 2000. However, some countries show a different trend. Despite continued investment from the government of Ethiopia as well as donors, CGF levels are still high in Ethiopia. This study aimed to assess trends in CGF and associated sociodemographic, economic and water, sanitation, and hygiene (WASH) factors from 2000 to 2016 in Ethiopia.

**Methods:**

Data were taken from four rounds of the Ethiopia Demographic and Health Survey (EDHS). Children aged between 0 to 59 months were included. CGF indicators were categorised based on height-for-age z-score (HAZ) < -2 Standard deviation (SD), weight-for-age z-score (WAZ) < -2 SD and weight-for-height z-score (WHZ) < -2 SD. CGF trends were estimated for predicted probabilities and odds ratios (ORs) between 2000 and 2016.

**Results:**

A total sample size of 31978 for HAZ, 32045 for WAZ and 32246 for WHZ were included in the current study. Stunting decreased from an adjusted odds ratio (AOR) = 0.77 (95% CI: 0.67 to 0.88) in 2005 to an AOR = 0.45 (95% CI: 0.39 to 0.53) in 2016 compared with the year 2000. Compared with data in 2000, underweight decreased from an AOR of 0.70 (95% CI: 0.61 to 0.80) in 2005 to an AOR of 0.43 (95% CI: 0.36 to 0.50) in 2016. Wasting declined from an AOR of 0.91 (95% CI: 0.75 to 1.10) in 2005 to an AOR of 0.76 (95% CI: 0.61 to 0.94) in 2016, compared with data in 2000.

**Conclusions:**

Between 2000 to 2016, there was a decline in CGF levels albeit the levels are still relatively high compared with the World Health Organization (WHO) cut-off levels for public health concern. Observed rates of change varied across sociodemographic, economic and WASH factors which suggest that interventions tailored towards addressing the imbalances across those factors are required.

## Introduction

Child growth can be affected by overall quality of living as well as access to basic needs, such as food, safe water, housing and healthcare services [1]. The primary causes of CGF (i.e. childhood infections, child undernutrition and food insecurity) are usually observed in regions with a high proportion of CGF [2]. Assessing CGF is not only useful for directly exploring morbidity, mortality and nutritional status but also for signaling long-term impacts such as impaired cognition and productivity in adulthood in high burden regions [3–7]. Since 1990, there has been a significant reduction in CGF globally, such that the prevalence of stunting, wasting and being underweight has reduced by 35%, 11% and 36%, respectively [6]. However, rapid demographic growth together with the presence of social, economic and political inequalities between population subgroups, poses additional challenges in regions like Africa [6].

Child growth failure manifested as under-5 stunting, wasting and being underweight, is a specific subset of child under-nutrition that excludes micronutrient deficiencies [8]. A child is considered to be stunted, wasted or underweight if his/her HAZ, WHZ, or WAZ each falls less than -2 SD below WHO growth reference standards for a healthy child population [9]. Globally in 2017, an estimated 22.2% of children under-5 were stunted, 7.5% were wasted, and 5.6% were underweight [1]. This burden was concentrated in LMICs, where almost all stunted, wasted or underweight children live [1, 10]. Children living in many LMICs have faced challenges due to extreme poverty, inadequate access to healthcare services, food insecurity, and inadequate WASH services [11]. Compared with the year 2000, by the end of 2015 most countries had lowered their rates of children who were stunted, wasted and underweight [12]. However, in some countries such as South Sudan, Chad, Ethiopia, Madagascar and Sudan, in all CGF indicators has not occurred [12].

Despite reductions in levels of undernutrition over the past 15 years [13], the public health impetus for ending CGF in Ethiopia has never been greater. In 2019, the Mini EDHS showed 37% of under-5 children were stunted, 21% were underweight and 7% were wasted at a national level [14] which are higher levels compared with the averages for developing countries of 25% for stunting and 8.9% for wasting [15]. There were also substantial regional variations in the prevalence of CGF in Ethiopia. For example, stunting was 47% in the Amhara region, 43% in Benishangul-Gumuz and 41% in Dire Dawa and Afar. Being underweight was the highest in Afar (36%), followed by Benishangul-Gumuz (34%) and Amhara (29%), while wasting was the highest in Somali (23%) and Afar (18%) [13].

Tackling CGF has been a priority of the Ethiopian Government through large-scale programs such as the Health Extension Program (HEP) to improve access to health services, the Enhanced Outreach Strategy, Targeted Supplementary Food and Community Management of Acute Malnutrition [16]. In 2008, these programs were integrated into the HEP and the first National Nutrition Strategy was launched. These programs were community based and aimed at providing education and/or supplementary foods. The implementation of this strategy occurred in two phases from 2008 to 2013 and then from 2013 to 2014 [16]. In 2015, Ethiopia declared the Seqota Declaration to end child malnutrition by 2030 by launching a Multi-Sectoral National Nutrition Program (NNP-II) aimed at guiding sectors and development partners in scaling-up nutrition interventions through the period from 2016 to 2020 [17]. Despite these programs, the country is lagging behind the expected trajectory needed to achieve the global nutrition target by 2025 and to end undernutrition by 2030.

Assessing patterns and trends of CGF indicators helps guide discussions about country-level progress and direct interventions to where they are most needed [5, 18, 19]. Although country-level estimates are useful for international comparison and benchmarking [12], they may mask disparities in CGF across sociodemographic, economic, and environmental factors. Knowing the level of detail at local levels will provide important insights into the stalled progress to eliminate CGF within the country. It is anticipated the current study findings will help to inform future policy and program decisions towards ending CGF in Ethiopia. As such, this study aimed to assess trends in CGF and associated sociodemographic, economic and WASH factors from 2000 to 2016 in Ethiopia.

## Methods

### Data type

Data for this study were obtained from four rounds of EDHS: 2000, 2005, 2011 and 2016. Each survey was conducted in Ethiopia at 5-year intervals in all nine regions and in two city jurisdictions. Note, the gap between 2000 and 2011 publication was 6 years, due to slight delays in publication of the 2011 report rather than delays in data collection. This indicates the publication of report was a bit longer for 2011 survey compared with others. EDHS is a national representative and large-scale survey. Many countries have conducted the multiple Demographic and Health Surveys (DHS) to establish trends that enable decision makers at the national levels to gauge progress. Children aged between 0 to 59 months and mothers/caregivers 15 to 49 years were included in this study. All children with valid anthropometric measurements were included.

### Sampling procedures of the EDHS

Two-stage stratified, and cluster random sampling techniques were used in the EDHS. In the first stage, enumeration areas or clusters were selected for each survey. The sampling frame used for the 2000 and 2005 EDHS was taken from the Population and Housing Census (PHC) which was conducted in 1994, while the 2011 and 2016 EDHS used the sampling frame from PHC 2007. Enumeration areas were selected from both urban and rural areas with a fixed number of households for each survey. Sociodemographic, economic, environmental and other disease related information for selected households were obtained by interviewing women. In addition, anthropometry was collected from children and from women who were interviewed. After identifying a nationally representative sample of households, a total of 64401 (sum of all surveys) eligible women aged 15–49 years were identified for individual interview. Of eligible women in each survey, the response rate was 97.8%, 95.6%, 95.0% and 94.6% in 2000, 2005, 2011 and 2016 EDHS, respectively. A pooled sample of the four surveys yielded an unweighted 43029 child-mother/caregiver pairs. In the current paper, we included 31978 children for HAZ, 32045 for WAZ and 32246 for WHZ analyses, respectively. The weighted sample yielded a total of 101415 children, including 29430 children in 2000, 12918 in 2005, 30120 in 2011 and 28947 in 2016. Detailed survey methods and sampling procedures are found in the respective EDHS reports [13, 20–22]. EDHS data were downloaded with permission from the DHS Program website using Integrated Public Use Microdata Series (IPUMS-DHS) extract system [23].

### Data preparation and statistical analyses

Child growth failure was classified using HAZ, WAZ and WHZ as defined by WHO 2006 standard and classifications [8, 9]. Child growth was considered as failure when child’s HAZ, WAZ or WHZ fell below −2 SD from the median of the WHO reference population for a given age. Explanatory variables included: age; sex and birth order of the child; age of mothers at birth; maternal and paternal education; household wealth quintiles; geographic region; area of residence; and access to WASH facilities.

We pooled data from four EDHS datasets. Prior to performing statistical analyses, we checked for missing observations and for any small cell frequencies through cross-tabulation between explanatory and outcome variables. Categories deemed to have small cell frequencies were merged in ways that preserved common sense meanings for each category. We have included sampling weight, clustering and stratification variables which were provided by DHS to account for the complex survey design. The STATA command ‘***svyset***’ was used to declare the survey design while all estimations were performed by using survey-specific command ‘***svy*.**’ A univariable logistic regression model was fitted with each explanatory variable to select candidates with p-values < 0.25 for the multivariable model. After which, a multivariable logistic regression model was fitted to estimate adjusted trends in ORs and predicted probabilities along with confidence intervals (CI). Interaction terms were fitted one at a time between survey year and each predictor variable. Threshold for statistical significance was declared at the outset as p < 0.05. All analyses were performed using STATA version 15.

## Results

### Overall prevalence of CGF by selected predictors

Table 1 presents the overall weighted sample (n) included in the analysis as well as prevalence of stunting, underweight and wasting across sociodemographic, economic and WASH factors. The overall prevalence of stunting (HAZ < -2 SD) was 57.45%, 50.85%, 44.34% and 38.39% in 2000, 2005, 2011 and 2016, respectively. The highest levels (48.81%) of child linear growth failure (stunting) were concentrated in rural areas. Stunting ranged from a high of 55.88% in the Amhara region to a low of 22.65% in Addis Ababa. About 50.38% of stunted children were born to mothers with no formal education which is in stark comparison with the 18.43% stunted children were born to mothers with higher educational. About 51.44% of all stunted children were found in the first wealth quintile and 50.32% were in the second wealth quintile. Children aged 24 to 59 months had a higher proportion of stunting (55.89%) compared with 0 to 5 months (15.73%) (Table 1).

**Table 1:** Overall prevalence of stunting, underweight and wasting among children aged 0–59 months in Ethiopia, EDHS 2000 to 2016.

Variable	n	Stunting (%)	n	Underweight (%)	n	Wasting (%)
**Survey year**						
2000	9810	5636 (57.45)	9810	4035 (41.13)	9810	1225 (12.49)
2005	4306	2189 (50.85)	4306	1429 (33.18)	4306	526 (12.21)
2011	10040	4452 (44.34)	10040	2892 (28.80)	10040	989 (9.85)
2016	9588	3681 (38.39)	9752	2314 (23.73)	9607	970 (10.09)
**Residence**						
Urban	3640	1263 (34.69)	3649	704 (19.29)	3639	270 (7.42)
Rural	30104	14695 (48.81)	30260	9966 (32.93)	30124	3439 (11.42)
**Region**						
Tigray	2299	1155 (50.23)	2307	779 (33.77)	2298	270 (11.76)
Afar	321	151 (47.10)	324	128 (39.56)	323	58 (17.92)
Amhara	7576	4234 (55.88)	7593	2855 (37.59)	7569	884 (11.68)
Oromia	14270	6245 (43.77)	14346	4085 (28.47)	14288	1497 (10.48)
Somali	919	322 (35.04)	929	311 (33.43)	929	205 (22.09)
Beni. Gumuz	357	166 (46.63)	359	126 (35.21)	357	46 (12.79)
SNNP	7127	3453 (48.45)	7172	2272 (31.68)	7126	689 (9.67)
Gambela	87	27 (31.58)	87	20 (23.09)	87	13 (14.79)
Harari	71	25 (35.57)	72	15 (21.17)	72	7 (9.38)
Addis Ababa	600	136 (22.65)	603	47 (7.85)	599	26 (4.34)
Dire Dawa	116	42 (36.47)	118	32 (26.91)	116	14 (12.26)
**Paternal education**						
No schooling	17835	9270 (51.98)	17938	6540 (36.46)	17877	2261 (12.65)
Primary	11619	5139 (44.23)	11659	3228 (27.68)	11594	1093 (9.42)
Secondary	2541	946 (37.24)	2556	547 (21.42)	2541	193 (7.61)
Higher	886	212 (23.93)	887	109 (12.27)	885	63 (7.09)
**Maternal education**						
No schooling	24597	12392 (50.38)	24704	8602 (34.82)	24623	2941 (11.94)
Primary	7398	3111 (42.05)	7451	1817 (24.38)	7399	658 (8.89)
Secondary	1353	382 (28.22)	1357	215 (15.86)	1346	85 (6.33)
Higher	396	73 (18.43)	397	35 (8.91)	395	26 (6.51)
**Maternal age**						
15–24	8096	3638 (44.94)	8135	2291 (28.16)	8094	922 (11.39)
25–34	3053	1399 (45.81)	3067	917 (29.89)	3048	325 (10.65)
35–44	1966	973 (49.50)	1981	672 (33.92)	1971	214 (10.84)
45–49	20630	9948 (48.22)	20725	6790 (32.76)	20650	2249 (10.89)
**Wealth quintile**						
Poorest	7586	3902 (51.44)	7654	2804 (36.64)	7610	1000 (13.15)
Poorer	7501	3775 (50.32)	7535	2646 (35.12)	7510	921 (12.27)
Middle	7021	3414 (48.62)	7037	2294 (32.60)	7022	795 (11.32)
Richer	6581	3068 (46.62)	6604	1859 (28.15)	6570	626 (9.52)
Richest	5055	1799 (35.58)	5079	1067 (21.00)	5052	367 (7.27)
**Sex of child**						
Male	17172	8457 (49.25)	17282	5728 (33.14)	17206	2063 (11.99)
Female	16572	7501 (45.26)	16627	4941 (29.72)	16557	1646 (9.94)
**Child age (months)**						
0–5	3436	541 (15.73)	3485	439 (12.59)	3409	496 (14.56)
6–23	10129	4139 (40.87)	10171	3057 (30.06)	10140	1628 (16.06)
24–59	20179	11278 (55.89)	20253	7174 (35.42)	20214	1585 (7.84)
**Birth order**						
First	6005	2682 (44.66)	6037	1644 (27.22)	6003	575 (9.58)
Second	5592	2518 (45.03)	5611	1563 (27.86)	5582	536 (9.60)
Third	4801	2257 (47.01)	4834	1566 (32.40)	4807	524 (10.90)
Fourth+	13439	6636 (49.38)	13513	4624 (34.22)	13456	1588 (11.80)
Water*						
Improved	17524	7987 (45.58)	17635	5276 (29.92)	17554	1878 (10.70)
Unimproved	15631	7689 (49.19)	15684	5193 (33.11)	15620	1765 (11.30)
Sanitation*						
Improved	3796	1454 (38.29)	3800	891 (23.46)	3795	319 (8.39)
Unimproved	29359	14222 (48.44)	29520	9577 (32.44)	29378	3325 (11.32)
Handwashing*						
Improved	19023	9608 (50.51)	19095	6487 (33.97)	19014	2213 (11.64)
Unimproved	14131	6069 (42.94)	14224	3982 (27.99)	14160	1431 (10.10)
WASH*						
Improved	1083	310 (27.51)	1088	180 (16.57)	1080	82 (7.54)
Unimproved	31346	15291 (47.95)	31505	10063 (31.94)	31367	3498 (11.15)

* Visitors were excluded; SNNP = Southern Nations, Nationalities and People; WASH = water, sanitation, and handwashing.

The percentage of children underweight (WAZ < -2 SD) was 41.13% in 2000, 33.18% in 2005, 28.80% in 2011and 23.73% in 2016. Of all underweight children, 39.56% were living in the Afar region. More than one in three (34.82%) underweight children were born to mothers with no education compared with 8.91% underweight children were born to mothers with higher educational status. As expected, the wealth quintile was inversely related to being underweight, with 36.64% in the lowest quintile and 21% in the highest quintile. Being underweight ranged from a high of 35.42% in children aged 24 to 59 months to a low of 12.59% in children aged 0 to 5 months. Overall, wasting (WHZ < -2 SD) in Ethiopia was 12.49%, 12.21%, 9.85% and 10.09% in 2000, 2005, 2011 and 2016, respectively. Wasting was highest in Somali (22.09%) while the lowest levels were observed in Addis Ababa (4.34%). The percentage of wasting was 13.15% in the lowest wealth quintile and 7.27% in the highest quintile (Table 1). S1 Fig shows geographical variation of CGF prevalence in Ethiopia between 2000 and 2016.

### Trends in child growth failure

**Fig 1:**
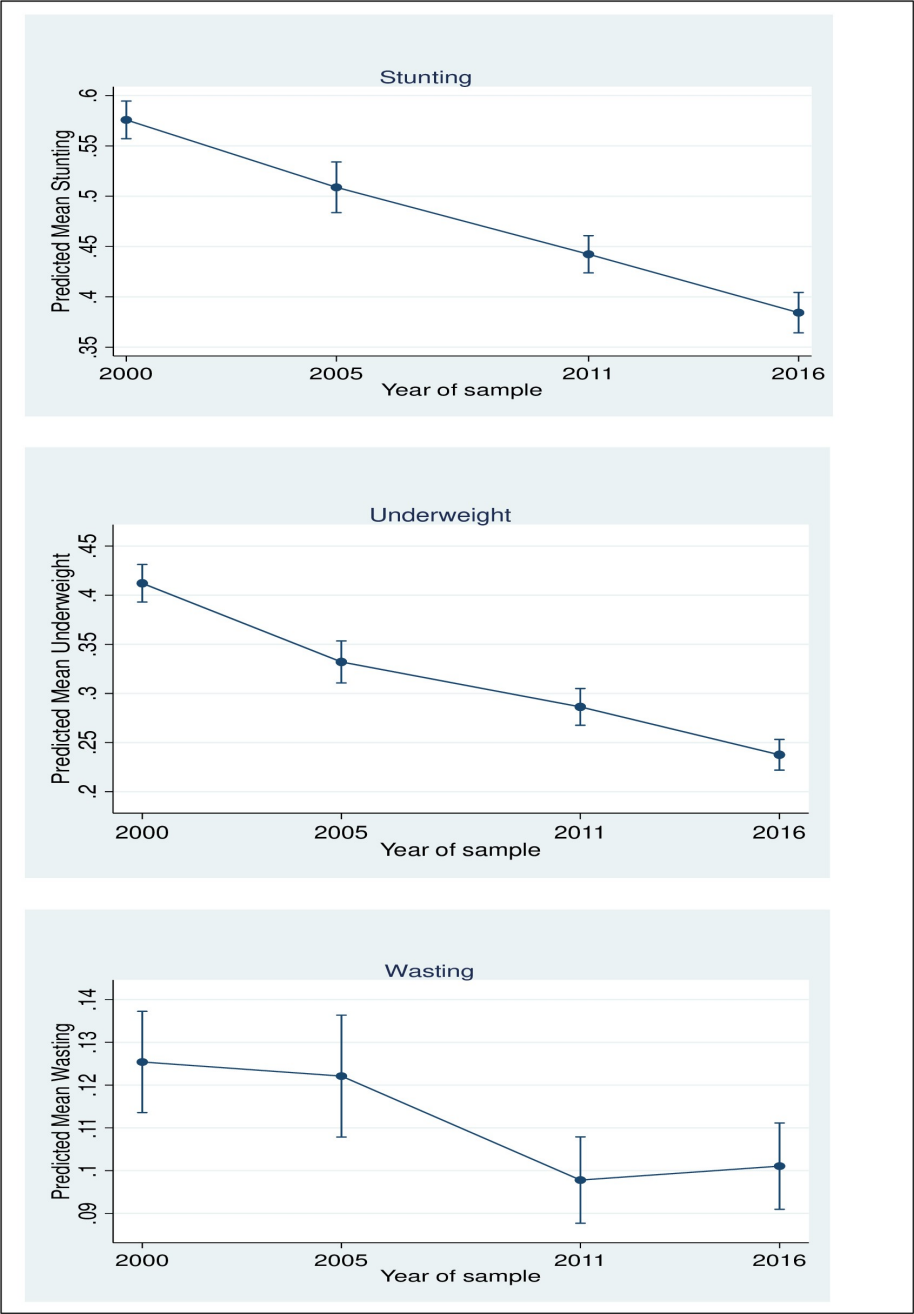
Predicted mean of CGF indicators among under-5 children in Ethiopia.

Fig 1 reveals a marked fall in the predicted mean of CGF indicators in Ethiopia between 2000 and 2016.

### Stunting

**Fig 2:**
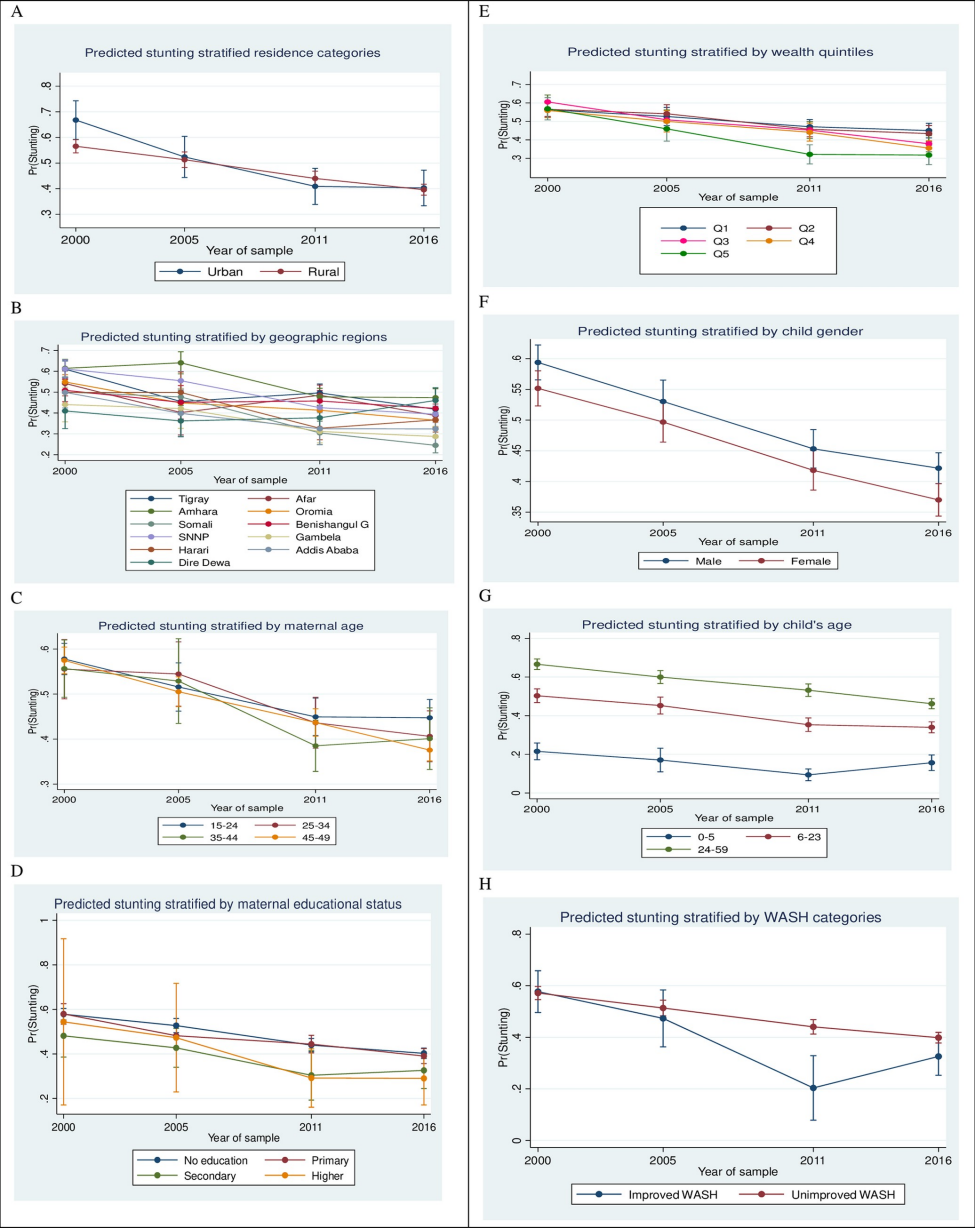
Adjusted predicted probabilities of stunting by key predictors among under-5 children in Ethiopia, EDHS 2000 to 2016.

The predicted probabilities of stunting have declined in urban-rural areas since 2000, and this decline was of a larger magnitude in rural than in urban areas. Predicted probabilities for stunting showed a marked decline in most geographic regions between 2000 and 2016. Some of the largest decreases were observed in the predicted probabilities between 2005 and 2011, for instance an 18-point decrease (i.e., 0.48 in 2005 to 0.30 in 2011) in the Somali region, a 17-point decrease in the Harari region and a 16-point decline in the Amhara region (S1 Table). The probability of stunting decreased for children of all paternal and maternal education categories between 2000 and 2011. There was a decrease in the predicted probability of stunting across all wealth quintiles between 2000 and 2016. However, stunting appeared static in the highest wealth quintile between 2011 and 2016. Fig 2E illustrates a wider disparity across wealth quintiles in the adjusted predicted probabilities of stunting between 2005 and 2016 compared with other time periods. The predicted probability of stunted children among households with access to improved drinking water sources and sanitation facilities declined between 2000 and 2011, and this decline was of the greatest magnitude between 2005 and 2011. Children who had access to combined improved WASH showed a larger decline of 27 points in the predicted probability of stunting (S1 Table) and there was a slight wide gap in trend lines between 2005 and 2011 (Fig 2H).

Stunting showed a statistically significant reduction with survey years (Table 2). In Ethiopia, compared with data from the 2000, the odds of being stunted showed a decreasing trend from an AOR of 0.77 (95% CI: 0.67 to 0.88) in 2005 to an AOR of 0.54 (95% CI: 0.44 to 0.66) in 2011 and to an AOR of 0.45 (95% CI: 0.39 to 0.53) in 2016. The odds of being stunted was highest in 2011 for rural (AOR = 1.88 with 95% CI: 1.23 to 2.88) compared with urban areas. Children in the Dire Dawa city jurisdiction had the highest odds of stunting compared with other regions in both 2011 (AOR = 1.90; 95% CI: 1.103 to 3.51) and 2016 (AOR = 2.71; 95% CI: 1.47 to 5.02).

**Table 2:** Trends in adjusted odds ratios for stunting, EDHS 2000 to 2016.

Category	2000		2005		2011		2016	
	AOR (95% CI)	p value	AOR (95% CI)	p value	AOR (95% CI)	p value	AOR (95% CI)	p value
**Survey year**	Ref		0.77 (0.67, 0.88)	< 0.001	0.54 (0.44, 0.66)	< 0.001	0.45 (0.39, 0.53)	< 0.001
**Residence**								
Urban	Ref		Ref		Ref		Ref	
Rural	1.64 (0.82, 1.98)	0.667	1.56 (0.96, 2.51)	0.070	1.88 (1.23, 2.88)	0.004	1.58 (1.01, 2.47)	0.045
**Region**								
Tigray	1.61 (1.06, 2.45)	0.025	0.78 (0.42,1.45)	0.434	1.33 (0.80, 2.19)	0.272	0.93 (0.57, 1.54)	0.789
Afar	1.10 (0.67, 1.80)	0.710	0.84 (0.39, 1.79)	0.649	1.71 (0.98, 2.97)	0.059	1.09 (0.63, 1.91)	0.751
Amhara	1.76 (1.13, 2.74)	0.012	1.78 (0.96, 3.28)	0.067	1.20 (0.73, 1.97)	0.481	1.18 (0.72, 1.92)	0.512
Oromia	1.27 (0.80, 2.03)	0.315	1.01 (0.56, 1.81)	0.978	1.22 (0.75, 1.98)	0.422	0.97 (0.61, 1.55)	0.896
Somali	1.09 (0.70, 1.71)	0.697	1.40 (0.72, 2.73)	0.318	0.89 (0.50, 1.58)	0.693	0.64 (0.38, 1.10)	0.108
Beni.Gum	1.10 (0.69, 1.75)	0.696	1.21 (0.62, 2.37)	0.573	1.75 (1.01, 3.04)	0.046	1.49 (0.88, 2.51)	0.135
SNNP	1.64 (1.05, 2.57)	0.031	1.20 (0.66, 2.17)	0.549	0.96 (0.57, 1.61)	0.870	0.83 (0.50,1.35)	0.446
Gambela	0.80 (0.50, 1.27)	0.335	1.42 (0.66, 3.06)	0.364	1.21 (0.65, 2.28)	0.549	1.07 (0.59, 1.95)	0.825
Harari	0.90 (0.62, 1.31)	0.590	1.56 (0.76, 3.22)	0.224	1.01 (0.58, 1.76)	0.960	1.22 (0.70, 2.13)	0.485
Dire Dawa	0.67 (0.46, 0.98)	0.039	1.25 (0.61, 2.56)	0.548	1.90 (1.03, 3.51)	0.040	2.71 (1.47, 5.02)	0.001
Addis Ababa	Ref		Ref		Ref		Ref	
**Paternal education**								
No schooling	1.61 (0.85, 3.04)	0.146	0.85 (0.28, 2.62)	0.780	1.42 (0.57, 3.55)	0.455	1.14 (0.57, 3.52)	0.451
Primary	1.47 (0.79, 2.76)	0.225	0.75 (0.24, 2.32)	0.615	1.32 (0.53, 3.27)	0.550	1.17 (0.47, 2.91)	0.734
Secondary	1.34 (0.73, 2.47)	0.356	0.65 (0.21, 2.03)	0.455	0.94 (0.35, 2.56)	0.909	1.34 (0.52, 3.47)	0.734
Higher	Ref		Ref		Ref		Ref	
**Maternal education**								
No schooling	2.24 (0.44, 11.4)	0.332	1.09 (0.15, 7.86)	0.933	1.72 (0.28, 10.41)	0.557	1.47 (0.24, 8.84)	0.675
Primary	2.14 (0.42, 10.9)	0.358	0.89 (0.12, 6.41)	0.905	1.74 (0.29, 10.51)	0.545	1.38 (0.23, 8.28)	0.724
Secondary	1.31 (0.25, 6.86)	0.746	1.08 (0.14, 8.13)	0.942	1.41 (0.22, 8.90)	0.718	1.58 (0.25, 10.15)	0.630
Higher	Ref		Ref		Ref		Ref	
**Maternal age (years)**								
15–24	Ref		Ref		Ref		Ref	
25–34	0.92 (0.69, 1.22)	0.835	1.26 (0.80, 2.0)	0.320	1.04 (0.69, 1.58)	0.835	0.92 (0.60, 1.42)	0.706
35–44	1.21 (0.82, 1.79)	0.332	1.17 (0.67, 2.04)	0.582	0.82 (0.56, 1.22)	0.332	0.89 (0.57, 1.42)	0.629
45–49	1.04 (0.82, 1.32)	0.728	0.97 (0.72, 1.31)	0.831	0.96 (0.77, 1.22)	0.728	0.73 (0.57, 0.94)	0.016
**Wealth quintile**								
Poorest	Ref		Ref		Ref		Ref	
Poorer	1.02 (0.84, 1.23)	0.862	1.06 (0.74, 1.52)	0.758	0.93 (0.70, 1.24)	0.632	0.92 (0.66, 1.29)	0.638
Middle	1.21 (0.97, 1.50)	0.091	0.77 (0.53, 1.10)	0.144	0.76 (0.55, 1.05)	0.098	0.60 (0.42, 0.86)	0.005
Richer	0.99 (0.81, 1.22)	0.964	0.91 (0.62, 1.33)	0.620	0.89 (0.65, 1.24)	0.501	0.67 (0.47, 0.94)	0.023
Richest	0.93 (0.67, 1.28)	0.641	0.73 (0.47, 1.13)	0.158	0.49 (0.33, 0.73)	< 0.001	0.53 (0.35, 0.80)	0.003
**Sex of child**								
Male	Ref		Ref		Ref		Ref	
Female	0.86 (0.77, 0.96)	0.008	1.04 (0.86, 1.27)	0.667	1.04 (0.86, 1.06)	0.706	0.96 (0.79, 1.16)	0.666
**Child age (months)**								
0–5	Ref		Ref		Ref		Ref	
6–23	0.70 (0.44, 1.13)	0.150	1.09 (0.63, 1.87)	0.762	1.42 (0.88, 2.29)	0.150	0.74 (0.48, 1.14)	0.174
24–59	0.67 (0.42, 1.05)	0.081	1.00 (0.59, 1.67)	0.990	1.50 (0.95, 2.36)	0.081	0.62 (0.41, 0.94)	0.026
**Birth order**								
First	0.78 (0.60, 1.02)	0.072	1.04 (0.74, 1.46)	0.808	1.28 (0.98, 1.68)	0.072	1.30 (0.97, 1.74)	0.075
Second	0.95 (0.74, 1.23)	0.712	0.96 (0.69, 1.33)	0.795	1.05 (0.82, 1.35)	0.712	1.18 (0.90, 1.54)	0.230
Third	0.91 (0.69, 1.20)	0.505	0.88 (0.63, 1.24)	0.467	1.10 (0.83, 1.44)	0.505	0.90 (0.67, 1.22)	0.506
Forth+	Ref		Ref		Ref		Ref	
**Water**								
Improved	0.96 (0.81, 1.13)	0.629	0.88 (0.68, 1.15)	0.347	0.94 (0.75, 1.17)	0.563	0.99 (0.78, 1.25)	0.939
Unimproved	Ref		Ref		Ref		Ref	
**Sanitation**								
Improved	1.08 (0.74, 1.58)	0.684	1.02 (0.64, 1.63)	0.922	0.54 (0.38, 0.78)	0.001	0.75 (0.52, 1.08)	0.119
Unimproved	Ref		Ref		Ref		Ref	
**Handwashing**								
Improved	n/a	n/a	n/a	n/a	0.64 (0.37, 1.13)	0.124	0.98 (0.83, 1.16)	0.839
Unimproved	Ref	Ref	Ref		Ref		Ref	
**WASH**								
Improved	0.65 (0.37, 1.12)	0.122	0.82 (0.46, 1.44)	0.489	0.29 (0.12, 0.70)	0.01	0.70 (0.44, 1.10)	0.124
Unimproved	Ref		Ref		Ref		Ref	

AOR = adjusted odds ratio; Ref = reference group.

The odds of being stunted decreased with increases in maternal age in years and it was statistically significant for mothers aged 45 to 49 (AOR = 0.73 with 95% CI: 0.57 to 0.94) in 2016. The odds of being stunted decreased with an increase in wealth quintiles; 0.49 times lower in the highest wealth quintile compared with the lowest quintile in 2011 (AOR = 0.49; 95% CI: 0.33 to 0.73) and 47% lower in 2016 (AOR = 0.53; 95% CI: 0.35 to 0.80). In 2000, the odds of being stunted were 14% lower among female children compared with their counterparts (AOR = 0.86; 95% CI: 0.77–0.96). In 2016, children aged 24 to 59 months were 0.62 times less likely to be stunted compared with infants aged 0 to 5 months (AOR = 0.62; 95% CI: 0.41 to 0.94). In 2011, children in households with access to improved sanitation were 46% less likely to be stunted compared with children in households without improved sanitation (AOR = 0.54; 95% CI: 0.38 to 0.78). Also in 2011, children with access to improved combined WASH facilities were 71% less likely to be stunted (AOR = 0.29; 95% CI: 0.12 to 0.70) compared with children without access to improved combined WASH (Table 2).

### Underweight

**Fig 3:**
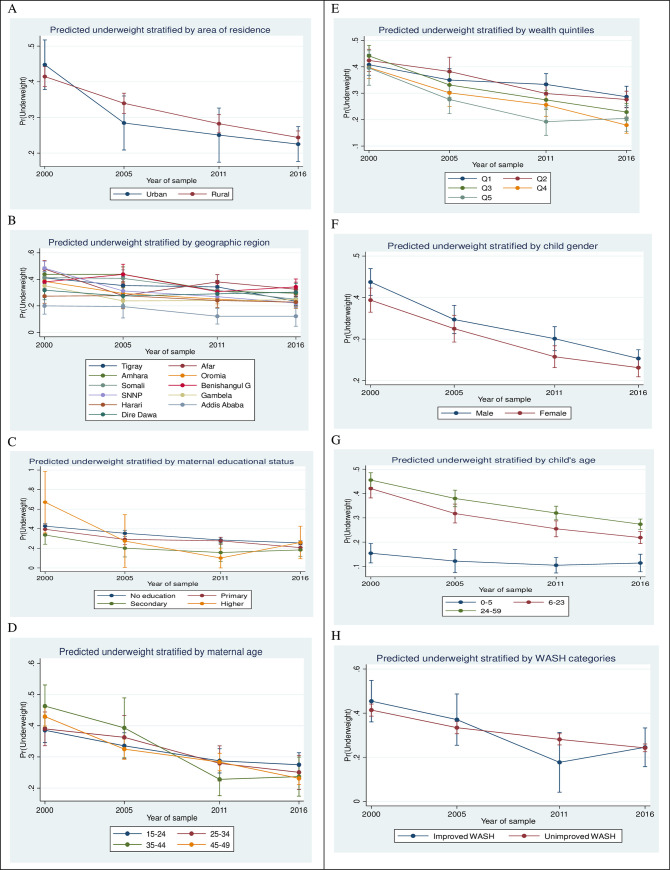
Adjusted predicted probabilities of being underweight by key predictors among under-5 children in Ethiopia, EDHS 2000 to 2016.

The predicted probability of being underweight has decreased by 17 points in urban areas (from 0.45 in 2000 to 0.28 in 2005) and by 7 points in rural areas (from 0.41 in 2000 to 0.34 in 2005) (S2 Table). The greatest decline in the predicted probabilities of underweight children between 2000 and 2005 was observed in the Afar region (20 points) followed by the SNNPR (17 points) and Gambela (11 points) compared with other regions. Between 2000 and 2011, being underweight decreased for children of all paternal and maternal education categories. The probability of being underweight has decreased across all wealth quintiles for all survey years (Fig 3E). The predicted probabilities of being underweight among households with access to improved drinking water sources declined from 0.42 in 2000 to 0.25 in 2016. A reduction in the predicted probability of being underweight for households with improved sanitation, reached the greatest change (16 points) between 2005 and 2011. Households with access to combined improved WASH facilities showed a larger decline in the predicted probability of children being underweight (19 points) from 0.37 in 2005 to 0.18 in 2011 (S2 Table).

The odds of being underweight showed a decreasing trend from AOR of 0.70 (95% CI: 0.61 to 0.80) in 2005 to 0.52 (95% CI: 0.43 to 0.65) in 2011 and to 0.43 (95% CI: 0.36 to 0.50) in 2016 compared with the year 2000 (Table 3). Compared to Addis Ababa, children in the Afar region had 0.41 times lower odds of being underweight (AOR = 0.41; 95% CI: 0.18 to 0.93) in 2005 while it was not statistically significant in other years. Similarly, children in the SNNP region had 0.48 times lower odds of being underweight in 2005 (AOR = 0.48; 95% CI: 0.24 to 0.95) compared with children in Addis Ababa jurisdiction. Between 2000 and 2011, the odds of being underweight decreased with increases in wealth quintiles. However, the trend was not consistent. For instance, in 2011 the odds of being underweight decreased from an AOR of 0.79 (95% CI: 0.60 to 1.03) in the lower quintile to an AOR of 0.49 (95% CI: 0.31 to 0.76) in the highest quintile. In the period 2005 to 2016, the odds of being underweight decreased for children aged 6 to 23 months from an AOR of 0.83 (95% CI: 0.47 to 1.49) in 2005 to an AOR of 0.53 (0.33 to 0.87) in 2016, as well as for children aged 24 to 59 months (from an AOR of 0.95 to an AOR of 0.62). There were higher odds of being underweight among children of first birth order (AOR = 1.50; 95% CI: 1.13 to 1.98) in 2011 and (AOR = 1.50; 95% CI: 1.12 to 2.01) in 2016 compared with children of fourth and over birth order. Compared with households with unimproved sanitation facilities, households with access to improved sanitation facilities had 52% lower odds of being underweight in 2000 (AOR = 0.70; 95% CI: 0.54 to 0.91) and in 2011 (AOR = 0.48; 95% CI: 0.32 to 0.72) while it was not statistically significant in other years (Table 3).

**Table 3:** Trends in adjusted odds ratios for children underweight, EDHS 2000 to 2016.

Category	2000		2005		2011		2016	
	AOR (95% CI)	p value	AOR (95% CI)	p value	AOR (95% CI)	p value	AOR (95% CI)	p value
**Survey year**	Ref		0.70 (0.61, 0.80)	<0.001	0.52 (0.43, 0.65)	<0.001	0.43 (0.36, 0.50)	<0.001
**Residence**								
Urban	Ref		Ref		Ref		Ref	
Rural	1.19 (0.77, 1.83)	0.437	1.52 (0.97, 2.36)	0.065	1.37 (0.92, 2.06)	0.126	1.29 (0.89, 1.86)	0.176
**Region**								
Tigray	0.74 (0.37, 1.50)	0.41	0.80 (0.39, 1.64)	0.538	1.34 (0.67, 2.71)	0.410	0.77 (0.35, 1.72)	0.525
Afar	0.83 (0.40, 1.75)	0.629	0.41 (0.18, 0.93)	0.034	1.20 (0.57, 2.51)	0.629	0.93 (0.40, 2.16)	0.859
Amhara	0.96 (0.49, 1.92)	0.918	1.04 (0.52, 2.07)	0.916	1.04 (0.52, 2.06)	0.918	0.95 (0.43, 2.11)	0.900
Oromia	1.05 (0.53, 2.09)	0.881	0.67 (0.34, 1.32)	0.248	0.95 (0.48, 1.89)	0.881	0.81 (0.37, 1.80)	0.611
Somali	0.85 (0.39, 1.85)	0.681	1.01(0.47, 2.16)	0.981	1.18 (0.54, 2.56)	0.681	0.83 (0.35, 1.96)	0.672
Ben. Gumuz	0.77 (0.36, 1.64)	0.496	1.33 (0.63, 2.81)	0.453	1.30 (0.61, 2.77)	0.496	1.54 (0.66, 3.60)	0.317
SNNP	1.46 (0.72, 2.96)	0.29	0.48 (0.24, 0.95)	0.036	0.68 (0.34, 1.38)	0.290	0.52 (0.23, 1.17)	0.114
Gambela	0.96 (0.44, 2.13)	0.927	0.58(0.27, 1.24)	0.159	1.04 (0.47, 2.30)	0.927	1.08 (0.44, 2.63)	0.869
Harari	0.65 (0.31, 1.37)	0.258	1.06 (0.45, 2.47)	0.897	1.53 (0.73, 3.20)	0.258	1.43 (0.61, 3.33)	0.410
Dire Dawa	0.62 (0.29, 1.33)	0.223	0.83 (0.38, 1.85)	0.651	1.60 (0.75, 3.41)	0.223	1.70 (0.70, 4.15)	0.242
Addis Ababa	Ref		Ref		Ref		Ref	
**Paternal education**								
No schooling	0.71 (0.31, 1.62)	0.418	1.83 (0.4, 7.14)	0.381	1.40 (0.62, 3.19)	0.418	1.13 (0.52, 2.46)	0.752
Primary	0.69 (0.31, 1.56)	0.376	1.82 (0.46, 7.21)	0.393	1.45 (0.64, 3.27)	0.376	1.01 (0.45, 2.22)	0.989
Secondary	1.05 (0.42, 2.60)	0.922	1.61 (0.41, 6.36)	0.496	0.96 (0.38, 2.38)	0.922	1.28 (0.53, 3.09)	0.581
Higher	Ref		Ref		Ref		Ref	
**Maternal education**								
No schooling	2.00 (1.02, 3.94)	0.045	1.44 (0.30, 6.79)	0.646	3.25 (0.94, 11.2)	0.062	1.04 (0.42, 2.55)	0.937
Primary	2.04 (1.03, 4.06)	0.042	1.10 (0.24, 5.05)	0.907	3.20 (0.93, 11.0)	0.064	0.74 (0.30, 1.83)	0.519
Secondary	1.06 (0.52, 2.16)	0.862	0.72 (0.14, 3.62)	0.693	1.60 (0.43, 5.91)	0.480	0.59 (0.22, 1.61)	0.303
Higher	Ref		Ref		Ref		Ref	
**Maternal age (years)**								
15–24	Ref		Ref		Ref		Ref	
25–34	0.96 (0.69, 1.33)	0.805	1.11 (0.71, 1.74	0.648	0.94 (0.62, 1.43)	0.774	0.86 (0.55, 1.34)	0.502
35–44	072 (0.50, 1.03)	0.075	0.93 (0.59,1.56)	0.785	0.51 (0.32, 0.82)	0.005	0.58 (0.35, 0.95)	0.030
45–49	0.98 (0.79, 1.21)	0.857	0.78 (0.60, 1.02)	0.073	0.81 (0.63, 1.04)	0.099	0.65 (0.49, 0.84)	0.001
**Wealth quintile**								
Poorest	Ref		Ref		Ref		Ref	
Poorer	0.95 (0.74, 1.22)	0.677	1.08 (0.77, 1.51)	0.657	0.79 (0.60, 1.03)	0.085	0.88 (0.64, 1.22)	0.446
Middle	0.73 (0.55, 0.96)	0.025	0.79 (0.57, 1.11)	0.180	0.65 (0.48, 0.87)	0.004	0.63 (0.45, 0.89)	0.008
Richer	0.53 (0.39, 0.72)	<0.001	0.83 (0.57, 1.20)	0.328	0.71 (0.50, 1.00)	0.052	0.56 (0.38, 0.81)	0.002
Richest	0.63 (0.43, 0.92)	0.018	0.74 (0.48, 1.12)	0.155	0.49 (0.31, 0.76)	0.001	0.67 (0.42, 1.05)	0.081
**Sex of child**								
Male	Ref		Ref		Ref		Ref	
Female	1.04 (0.86, 1.25)	0.708	1.09 (0.87, 1.37)	0.448	0.96 (0.80, 1.16)	0.708	1.07 (0.87, 1.30)	0.518
**Child age (months)**								
0–5	Ref		Ref		Ref		Ref	
6–23	1.39 (0.84, 2.30)	0.205	0.83 (0.47, 1.49)	0.538	0.72 (0.43, 1.20)	0.205	0.53 (0.33 0.87)	0.013
24–59	1.16 (0.73, 1.84)	0.531	0.95 (0.55, 1.66)	0.867	0.86 (0.54, 1.37)	0.531	0.62 (0.39, 1.00)	0.048
**Birth order**								
First	98 (0.78, 1.22)	0.827	1.16 (0.83, 1.61)	0.385	1.50 (1.13, 1.98)	0.004	1.50 (1.12, 2.01)	0.007
Second	0.85 (0.69, 1.05)	0.138	1.02 (0.73, 1.41)	0.928	1.16 (0.89, 1.52)	0.261	1.28 (0.96, 1.71)	0.090
Third	1.14 (0.93, 1.39)	0.200	1.08 (0.79, 1.47)	0.633	1.26 (0.97, 1.64)	0.081	0.99 (0.76, 1.32)	0.984
Forth+	Ref		Ref		Ref		Ref	
**Water**								
Improved	0.99 (0.76, 1.28)	0.932	0.98 (0.75, 1.28)	0.878	1.01 (0.78, 1.31)	0.932	1.06 (0.83, 1.37)	0.628
Unimproved	Ref		Ref		Ref		Ref	
**Sanitation**								
Improved	0.70 (0.54, 0.91)	0.008	0.85 (0.51, 1.41)	0.529	0.48 (0.32, 0.72)	< 0.001	0.65 (0.41, 1.02)	0.061
Unimproved	Ref		Ref		Ref		Ref	
**Handwashing**								
Improved	n/a	n/a	n/a	n/a	0.67 (0.30, 1.49)	0.325	0.91 (0.76, 1.08)	0.268
unimproved	n/a	n/a	n/a	n/a	Ref		Ref	
**WASH**								
Improved	0.54 (0.20, 1.42)	0.208	0.99 (0.54, 1.82)	0.981	0.45 (0.16, 1.25)	0.124	0.85 (0.47, 1.53)	0.588
Unimproved	Ref		Ref		Ref		Ref	

AOR = adjusted odds ratio; Ref = reference group.

### Wasting

**Fig 4:**
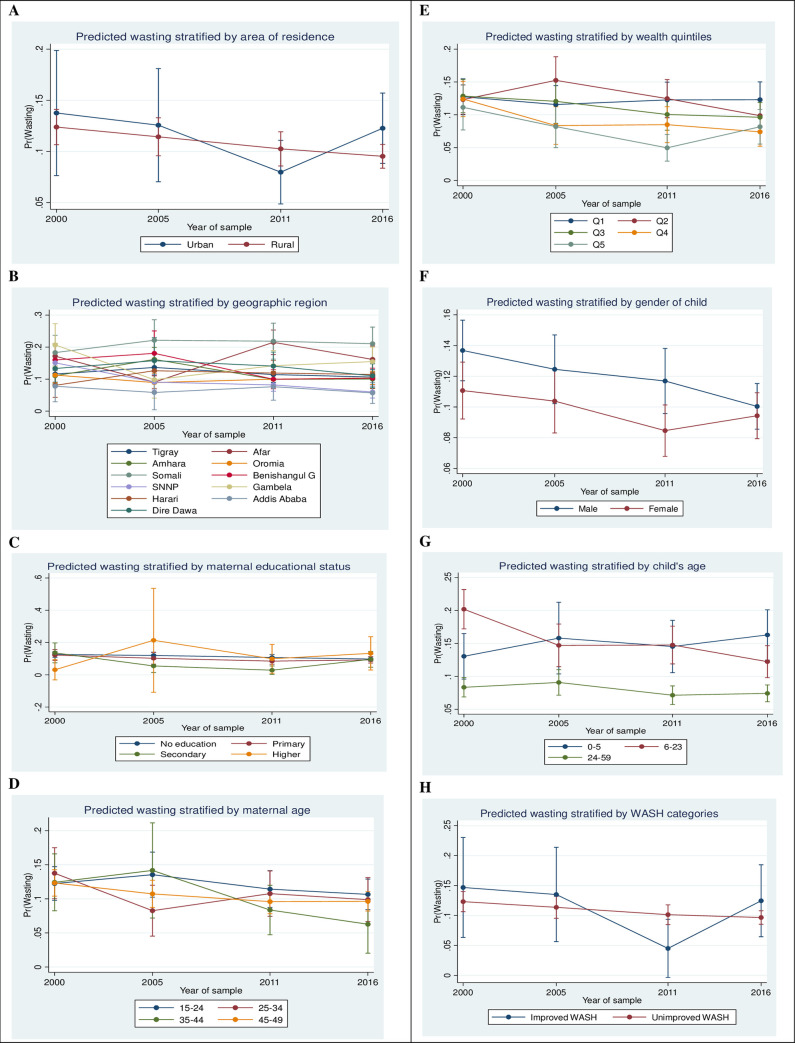
Adjusted predicted probabilities of wasting by key predictors among under-5 children in Ethiopia, EDHS 2000 to 2016.

The predicted probability of wasting in the SNNP region significantly declined between 2000 and 2016 compared with other regions. Gambela region had the largest reduction in predicted probability of wasting from 0.21 in 2000 to 0.10 in 2005 compared with other regions (S3 Table). Fig 4E shows there has been no marked fall in the predicted probabilities of wasting across wealth quintiles and WASH factors between 2000 and 2016.

The odds of being wasted showed a downward trend from an AOR of 0.91 (95% CI: 0.75 to 1.10) in 2005 to 0.76 (95% CI: 0.61 to 0.94) in 2016, compared with the year 2000 (Table 4). The probability of wasting decreased with increases in maternal education level, from an AOR of 1.09 for no education to an AOR of 0.26 for secondary education in 2000, and from an AOR of 0.11 for no education to 0.04 in 2005 for secondary education. The odds of wasting decreased with increases in wealth quintiles in 2000 and 2011 from an AOR of 1.02 vs 1.06 in the second quintile to an AOR of 0.37 vs 0.43 in the highest quintile, respectively. Although not consistent, the odds of wasting decreased from an AOR of 0.81 (95% CI of 0.51 to 1.28) in the second quintile to an AOR of 0.74 in the highest quintile in 2016. In 2016, girls were 0.70 times less likely to be wasted compared with boys (AOR = 0.70; 95% CI: 0.54 to 0.90). The odds of wasting decreased across survey years for children aged 6 to 23 months, from an AOR of 1.02 (95% CI: 0.71 to 1.47) in 2000 to an AOR of 0.42 (95% CI: 0.26 to 0.68) in 2016 (Table 4).

**Table 4:** Trends in adjusted odds ratios for wasting, EDHS 2000 to 2016.

Category	2000		2005		2011		2016	
	AOR (95% CI)	p value	AOR (95% CI)	p value	AOR (95% CI)	p value	AOR (95% CI)	p value
**Survey year**	Ref		0.91 (0.75, 1.10)	0.333	0.79 (0.59, 1.06)	0.110	0.76 (0.61, 0.94)	0.012
**Residence**								
Urban	Ref		Ref		Ref		Ref	
Rural	0.66 (0.36, 1.24)	0.198	1.01 (0.51, 2.01)	0.968	1.50 (0.81, 2.80)	0.198	0.85 (0.47, 1.51)	0.573
**Region**								
Tigray	0.99 (0.40, 2.48)	0.990	1.66 (0.49, 5.61	0.415	1.01 (0.40, 2.51	0.990	1.27 (0.53, 3.05	0.588
Afar	0.73 (0.29, 1.84)	0.508	0.65 (0.19, 2.20	0.484	1.36 (0.54, 3.43	0.508	1.30 (0.52, 3.24	0.573
Amhara	1.10 (0.44, 2.78)	0.834	2.15 (0.65, 7.06	0.208	0.91 (0.36, 2.28	0.834	1.25 (0.53, 2.99	0.610
Oromia	1.12 (0.45, 2.79)	0.800	1.05 (0.32, 3.50	0.932	0.89 (0.36, 2.21	0.800	1.27 (0.53, 3.05	0.586
Somali	0.78 (0.29, 2.08)	0.613	1.76 (0.50, 6.12	0.377	1.29 (0.48, 3.45	0.613	1.68 (0.66, 4.32	0.279
Ben. Gumuz	1.70 (0.65, 4.50)	0.282	1.59 (0.44, 5.70	0.475	0.59 (0.22, 1.55	0.282	0.84 (0.33, 2.14	0.707
SNNP	1.98 (0.79, 4.96)	0.145	0.76 (0.23, 2.51	0.650	0.51 (0.20, 1.27	0.145	0.49 (0.20, 1.23	0.128
Gambela	1.57 (0.56, 4.36)	0.389	0.56 (0.14, 2.19	0.402	0.64 (0.23, 1.77	0.389	0.97 (0.37, 2.53	0.952
Harari	0.63 (0.22, 1.77)	0.381	2.28 (0.60, 8.70	0.228	1.59 (0.56, 4.47	0.381	2.08 (0.73, 5.91	0.168
Dire Dawa	0.92 (0.35, 2.41)	0.858	1.68 (0.45, 6.30	0.444	1.09 (0.41, 2.88	0.858	1.13 (0.43, 2.98	0.810
Addis Ababa	Ref		Ref		Ref		Ref	
**Paternal education**								
No schooling	2.15 (1.04, 4.44)	0.038	1.12 (0.23, 5.48)	0.889	2.57 (0.76, 8.76)	0.131	1.24 (0.40, 3.90)	0.709
Primary	1.87 (0.91, 3.83)	0.089	1.11 (0.22, 5.56)	0.896	2.64 (0.77, 9.06)	0.123	1.34 (0.42, 4.29)	0.617
Secondary	0.95 (0.39, 2.29)	0.901	1.19 (0.22, 6.59)	0.838	1.57 (0.38, 6.48)	0.529	1.90 (0.52, 6.89)	0.331
Higher	Ref		Ref		Ref		Ref	
**Maternal education**								
No schooling	1.09 (0.39, 3.08)	0.865	0.11 (0.01, 1.84)	0.124	0.24 (0.02, 2.37)	0.221	0.15 (0.02, 1.41)	0.097
Primary	0.84 (0.30, 2.36)	0.737	0.09 (0.01, 1.63)	0.104	0.19 (0.02, 1.90)	0.157	0.15 (0.02, 1.39)	0.095
Secondary	0.26 (0.07, 0.98)	0.047	0.04 (0.00, 0.85)	0.039	0.05 (0.00, 0.65)	0.022	0.14 (0.01, 1.50)	0.103
Higher	Ref		Ref		Ref		Ref	
**Maternal age (years)**								
15–24	Ref		Ref		Ref		Ref	
25–34	1.23 (0.72, 2.12)	0.452	0.49 (0.26, 0.94)	0.031	0.81 (0.47, 1.40)	0.452	0.80 (0.46, 1.40)	0.430
35–44	1.45 (0.77, 2.74)	0.248	1.0 (0.48, 2.23)	0.923	0.69 (0.37, 1.30)	0.248	0.54 (0.23, 1.28)	0.165
45–49	1.23 (0.85, 1.78)	0.265	0.75 (0.51, 1.12)	0.161	0.81 (0.56, 1.17)	0.265	0.88 (0.62, 1.25)	0.483
**Wealth quintile**								
Poorest	Ref		Ref		Ref		Ref	
Poorer	1.02 (0.77, 1.35)	0.895	1.45 (0.91, 2.30)	0.120	1.06 (0.71, 1.58)	0.770	0.81 (0.51, 1.28)	0.364
Middle	0.79 (0.57, 1.11)	0.174	1.04 (0.63, 1.71)	0.879	0.79 (0.51, 1.23)	0.290	0.75 (0.47, 1.20)	0.225
Richer	0.66 (0.44, 0.98)	0.040	0.71 (0.41, 1.24)	0.234	0.68 (0.41, 1.13)	0.136	0.58 (0.34, 0.98)	0.042
Richest	0.37 (0.23, 0.59)	<0.001	0.79 (0.43, 1.46)	0.455	0.43 (0.24, 0.77)	0.005	0.74 (0.42, 1.29)	0.285
**Sex of child**								
Male	Ref		Ref		Ref		Ref	
Female	1.13 (0.84, 1.52)	0.430	0.89 (0.71, 1.12)	0.339	0.83 (0.60, 1.15)	0.258	0.70 (0.54, 0.90)	0.006
**Child age (months)**								
0–5	Ref		Ref		Ref		Ref	
6–23	1.02 (0.71, 1.47)	0.921	0.54 (0.31, 0.93)	0.028	0.60 (0.37, 0.98)	0.040	0.42 (0.26, 0.68)	0.001
24–59	0.88 (0.55, 1.42)	0.603	0.87 (0.50, 1.54)	0.643	0.74 (0.47, 1.18)	0.208	0.67 (0.42, 1.09)	0.108
**Birth order**								
First	0.85 (0.55, 1.32)	0.475	1.17 (0.72, 1.92)	0.524	1.17 (0.76, 1.83)	0.475	1.05 (0.69, 1.62)	0.813
Second	0.89 (0.61, 1.31)	0.564	1.23 (0.79, 1.91)	0.366	1.12 (0.76, 1.65)	0.564	0.67 (0.44, 1.01)	0.058
Third	0.98 (0.66, 1.45)	0.920	1.08 (0.70, 1.66)	0.723	1.02 (0.69, 1.51)	0.920	1.11 (0.73, 1.69)	0.625
Forth+	Ref		Ref		Ref		Ref	
**Water**								
Improved	0.97 (0.70, 1.34)	0.844	1.06 (0.75, 1.51)	0.729	1.03 (0.75, 1.43)	0.844	0.86 (0.62, 1.20)	0.376
Unimproved	Ref		Ref		Ref		Ref	
**Sanitation**								
Improved	1.55 (0.85, 2.82)	0.149	0.73 (0.36, 1.48)	0.379	0.64 (0.35, 1.17)	0.149	0.92 (0.51, 1.66)	0.779
Unimproved	Ref		Ref		Ref		Ref	
**Handwashing**								
Improved	n/a	n/a	n/a	n/a	0.48 (0.10, 2.32)	0.364	1.08 (0.84, 1.38)	0.556
Unimproved	n/a	n/a	n/a	n/a	Ref		Ref	
**WASH**								
Improved	0.41 (0.13, 1.32)	0.134	0.99 (0.40, 2.48)	0.989	0.33 (0.09, 1.23)	0.098	1.09 (0.47, 2.50)	0.843
Unimproved	Ref		Ref		Ref		Ref	

AOR = adjusted odds ratio; Ref = reference group.

## Discussion

This paper showed evidence of declining trends in child growth failure at a national level between 2000 and 2016 in Ethiopia. The decline was greatest between 2005 and 2011 compared with other periods. Possible reasons for the difference in the rate of decline need to be considered. Many factors may have contributed to the overall decline, including economic growth [24, 25] as well as the implementation of large-scale programs such as the Health Extension Program to improve access to health services, the Enhanced Outreach Strategy, Targeted Supplementary Food and the Community Management of Acute Malnutrition program [16]. However, a systematic review and meta-analysis which pooled studies conducted in various parts of Ethiopia failed to find a statistically significant association between economic growth and a reduction in child undernutrition [26]. On the other hand, evidence indicates that low family income and a poor living environment increases the risk of CGF, mainly due to inadequate access to health care as well as living in contaminated environments [26–28].

Although the downward trends in CGF are encouraging, the level of child growth failure in Ethiopia continues to be higher than the WHO cut-off levels for public health concern [29]. The international definition of ‘normal growth’ defines the first threshold, which includes 2.3% (very low prevalence of CGF) of the area under the normalized distribution [30]. As such 2.3% is used to establish subsequent thresholds for public health concern. In the current study, the decline in stunting was greater than the decline in children who met criteria for underweight or wasting. While the decline in rates of stunting in Ethiopia was comparable with other countries [31, 32], such declines in CGF indicators were not equally distributed across the country.

The decline in all CGF indicators between 2000 and 2016 was relatively rapid in urban areas compared with rural areas in Ethiopia. Previous studies have found that urban children had lower odds of stunting and being underweight compared with their rural counterparts [33, 34]. The observed association with urban areas could be attributed to better socioeconomic conditions, food security, maternal prenatal and postnatal care, access to quality complementary feeding, child immunization, WASH conditions, and fewer livestock resulting in cleaner environments and/or fewer infections. Region was another factor related to an unequal distribution in CGF indicators.

Since 2000, the predicted probabilities of having stunted children declined in all regions except in the Northern (Tigray and Afar between 2005 and 2011) and Eastern regions Harari and Dire Dawa. The analysis of trends in odds ratios also showed increased odds of stunting in Dire Dawa compared with other regions. The observed association could be attributed to regular drought conditions in the eastern part of Ethiopia [35]. Drought which causes water scarcity and crop failure would have negative impacts on food security and socioeconomic conditions in these areas. The current study shows a larger burden of stunting in the northern regions of the country which is consistent with previous studies [36–38]. The observed hotspots of stunting in the northern parts of the country are likely attributable to difference in child feeding practices [39, 40] and nutrition knowledge of mothers or caregivers [37]. Also, increased rates of stunting in some areas is likely attributable to climate variability in zones that were dependent on rain-fed agriculture [36, 41–43], which has an impact on household food security.

In addition to the downward trend in the adjusted predicted probabilities, the odds ratios indicated non-linear decreasing in stunting, underweight and wasting with an increase in maternal years of education. Previous studies have shown a statistically significant association between the education level of maternal/caregivers with child stunting [26, 36, 44, 45], underweight children [46] and child wasting [26]. These associations may be attributable to educated mothers/caregivers having greater access to and use of health services, healthier feeding behaviours and greater household income [47, 48]. In the current study, the odds of stunting decreased with increases in maternal age, which is a similar result to a previous study that found children of mothers older than 20–24 years of age had a lower probability of stunting compared with younger mothers [49]. A possible explanation for this is that women with more life experience may be more aware of, and have greater capacity to be, meeting the needs of their children, through the provision of adequate food and health care practices, which were not assessed in the current study [50–52]. CGF showed variation across household wealth quintiles between 2000 and 2016. The decline in predicted probabilities by wealth quintiles was higher for child stunting and being underweight compared with child wasting. Wealthier households showed a greater reduction in the odds of stunting and being underweight compared with households in lower wealth quintiles. This finding is in agreement with previous studies [27, 53–56].

There was a notable difference in CGF indicators between boys and girls. Although the predicted probabilities of child stunting declined similarly for boys and girls between 2000 and 2016, the odds ratios showed consistent decreases in wasting for girls compared with boys. A possible explanation for these variations are the differences in morbidity rates between girls and boys, as well as having a higher proportion of preterm births in boys compared with girls [57–59]. For instance, poor diet leads to an increased risk of infections, and an infection has profound effects on child nutritional status. This shows a vicious cycle between infections and wasting. A severe infection can cause wasting in children which potentially leads to a loss of appetite [60]. As wasting aggravates, children become more vulnerable to infections [60]. There were also CGF differences by child age and WASH facilities.

Increased odds of stunting, being underweight and wasting were associated with increased age of children, which is consistent with Hagos et al. [36] and Nguyen et al. [61] for stunting. Available evidence shows that lower risk at the younger age may be due to the protective effects of being breastfed which mostly continues during the first year of life [61–63]. CGF appears to develop during the period of weaning and then rises sharply at a later age which may be due to cumulative undernutrition associated with poor child feeding practices, inadequate complementary food, household conditions, household food insecurity and interacting with unsafe environment [32, 64–66].

The predicted probabilities of being stunted or underweight between 2000 and 2011 decreased with access to improved water, sanitation and combined WASH facilities. This may be attributable to large-scale investments by the Ethiopian government in sanitation during these times which may have plausibly improved child growth [16, 24, 26, 67–69]. The current findings have shown that sanitation alone and integrated improved WASH facilities were associated with decreased odds of stunting, while improved sanitation facilities alone were associated with decreased odds of children being underweight. Earlier studies found that access to improved WASH, either combined and/or individual components of WASH facilities, contributed to the reduction in child growth failure [26, 45, 70–75].

The current study used four large population-based surveys with large sample sizes that enable the findings to be representative of Ethiopia. However, the study had some limitations. As is often the situation in observational studies, the current study may be susceptible to recall and measurement biases. In the present study, it is difficult to make causal inferences and investigate temporal relationships between study outcomes and predictors. Unmeasured or residual confounding factors may significantly change the current estimates.

## Conclusions

This study shows that while CGF levels have decreased between 2000 and 2016 in Ethiopia, these levels remain high. CGF levels in the country shows substantial variation across sociodemographic, economic, and environmental characteristics, indicating benefits for some groups more than others. This study provides insights into trends in prevalence, predicted probabilities and determinants to inform policy makers to focus on target populations with relevant intervention programmes.

## Supporting information

S1 Fig Geographical variation of CGF in Ethiopia between 2000 and 2016. (TIF)

S1 Table Predicted probabilities for stunting over the four survey years, EDHS. (DOCX)

S2 Table Predicted probabilities for underweight over the four survey years, EDHS. (DOCX)

S3 Table Predicted probabilities for wasting over the four survey years, EDHS. (DOCX)
