# Supplementary figures and images for: Trends in child growth failure among children under five years of age in Ethiopia: Evidence from the 2000 to 2016 Demographic and Health Surveys
Source: PLoS One. 2021 Aug 5;16(8):e0254768. doi: 10.1371/journal.pone.0254768 (PMC8341490; doi:10.1371/journal.pone.0254768)

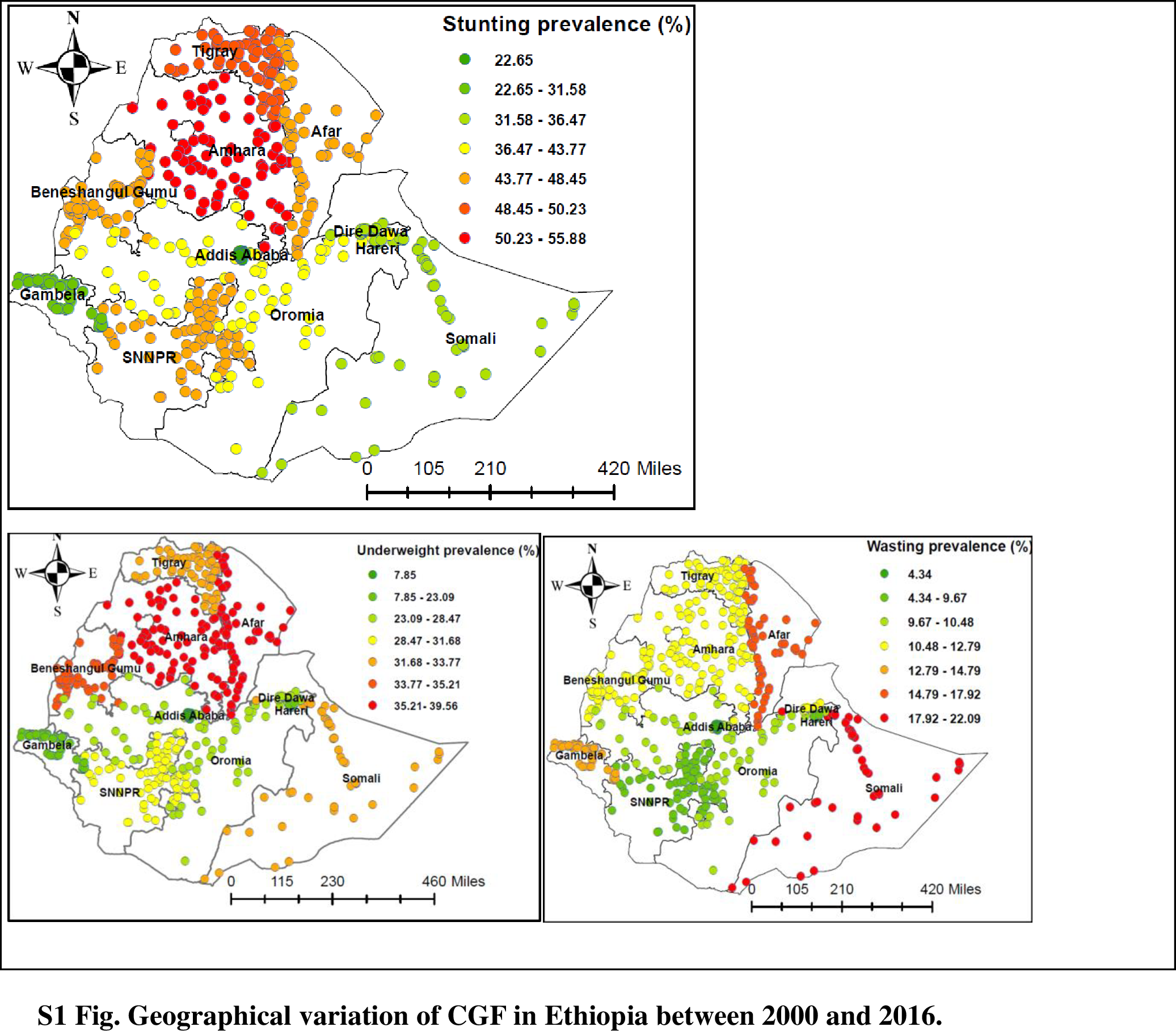

Supplement: S1 Fig — (TIF) [file pone.0254768.s001.tif]
