# Supplementary material for: Trends in child growth failure among children under five years of age in Ethiopia: Evidence from the 2000 to 2016 Demographic and Health Surveys
Source: PLoS One. 2021 Aug 5;16(8):e0254768. doi: 10.1371/journal.pone.0254768 (PMC8341490; doi:10.1371/journal.pone.0254768)
Supplement: S1 Table — (DOCX) [file pone.0254768.s002.docx]

**S1 Table. Predicted probabilities for stunting over the four survey years, EDHS.**

| **Variable** | **Category** | **2000** | **2005** | **2011** | **2016** |
| --- | --- | --- | --- | --- | --- |
|  |  | **(95% CI)** | **(95% CI)** | **(95% CI)** | **(95% CI)** |
| Residence | Urban | 0.67 (0.59, 0.74) | 0.53 (0.44, 0.61) | 0.41 (0.34, 0.48) | 0.41 (0.34, 0.48) |
|  | Rural | 0.56 (0.54, 0.59) | 0.51 (0.48, 0.54) | 0.44 (0.41, 0.47) | 0.40 (0.37, 0.42) |
| Region | Tigray | 0.61 (0.57, 0.65) | 0.45 (0.39, 0.52) | 0.50 (0.45, 0.54) | 0.42 (0.37, 0.47) |
|  | Afar | 0.54 (0.48, 0.60) | 0.40 (0.29, 0.51) | 0.48 (0.44, 0.53) | 0.39 (0.33, 0.44) |
|  | Amhara | 0.61 (0.57, 0.66) | 0.64 (0.59, 0.69) | 0.48 (0.44, 0.52) | 0.48 (0.43, 0.52) |
|  | Oromia | 0.55 (0.51, 0.58) | 0.45 (0.40, 0.50) | 0.41 (0.37, 0.45) | 0.36 (0.33, 0.40) |
|  | Somali | 0.50 (0.44, 0.56) | 0.48 (0.40, 0.55) | 0.30 (0.25, 0.36) | 0.24 (0.21, 0.28) |
|  | Beni. Gumuz | 0.51 (0.45, 0.56) | 0.45 (0.37, 0.53) | 0.46 (0.41, 0.51) | 0.42 (0.37, 0.47) |
|  | SNNP | 0.61 (0.57, 0.66) | 0.56 (0.51, 0.60) | 0.43 (0.39, 0.47) | 0.40 (0.36, 0.44) |
|  | Gambela | 0.44 (0.36, 0.52) | 0.42 (0.32, 0.51) | 0.31 (0.26, 0.37) | 0.29 (0.24, 0.34) |
|  | Harari | 0.50 (0.44, 0.56) | 0.50 (0.40, 0.60) | 0.33 (0.27, 0.38) | 0.37 (0.31, 0.42) |
|  | Addis Ababa | 0.50 (0.43, 0.58) | 0.40 (0.29, 0.51) | 0.33 (0.25, 0.41) | 0.33 (0.25, 0.41) |
|  | Dire Dawa | 0.41 (0.33, 0.50) | 0.36 (0.29, 0.43) | 0.38 (0.32, 0.44) | 0.46 (0.40, 0.52) |
| Paternal education | No schooling | 0.58 (0.55, 0.60) | 0.53 (0.50, 0.56) | 0.45 (0.41, 0.48) | 0.42 (0.39, 0.44) |
|  | Primary | 0.58 (0.55, 0.61) | 0.51 (0.45, 0.56) | 0.44 (0.40, 0.47) | 0.38 (0.35, 0.41) |
|  | Secondary | 0.57 (0.51, 0.62) | 0.46 (0.38, 0.53) | 0.35 (0.26, 0.43) | 0.39 (0.33, 0.46) |
|  | Higher | 0.56 (0.38, 0.73) | 0.55 (0.36, 0.73) | 0.35 (0.25, 0.45) | 0.32 (0.23, 0.42) |
| Maternal education | No schooling | 0.58 (0.55, 0.60) | 0.53 (0.50, 0.56) | 0.44 (0.41, 0.47) | 0.40 (0.38, 0.43) |
|  | Primary | 0.58 (0.53, 0.63) | 0.48 (0.43, 0.53) | 0.44 (0.40, 0.48) | 0.39 (0.36, 0.42) |
|  | Secondary | 0.48 (0.39, 0.58) | 0.43 (0.34, 0.51) | 0.30 (0.19, 0.42) | 0.33 (0.24, 0.41) |
|  | Higher | 0.54 (0.17, 0.92) | 0.47 (0.23, 0.72) | 0.29 (0.16, 0.42) | 0.29 (0.17, 0.41) |
| Maternal age | 15 - 24 | 0.58 (0.54, 0.61) | 0.52 (0.46, 0.57) | 0.45 (0.41, 0.49) | 0.45 (0.41, 0.49) |
|  | 25 - 34 | 0.56 (0.49, 0.62) | 0.54 (0.47, 0.62) | 0.44 (0.38, 0.49) | 0.41 (0.35, 0.46) |
|  | 35 - 44 | 0.56 (0.49, 0.62) | 0.53 (0.43, 0.62) | 0.39 (0.33, 0.44) | 0.40 (0.33, 0.47) |
|  | 45 - 49 | 0.57 (0.55, 0.60) | 0.51 (0.47, 0.54) | 0.44 (0.41, 0.47) | 0.38 (0.35, 0.40) |
| Wealth quintile | Poorest | 0.56 (0.52, 0.60) | 0.53 (0.48, 0.58) | 0.47 (0.43, 0.51) | 0.45 (0.41, 0.49) |
|  | Poorer | 0.56 (0.53, 0.60) | 0.54 (0.49, 0.59) | 0.46 (0.42, 0.50) | 0.43 (0.39, 0.48) |
|  | Middle | 0.60 (0.57, 0.64) | 0.51 (0.45, 0.56) | 0.45 (0.41, 0.50) | 0.38 (0.34, 0.42) |
|  | Richer | 0.56 (0.52, 0.60) | 0.50 (0.44, 0.56) | 0.44 (0.39, 0.49) | 0.36 (0.32, 0.40) |
|  | Richest | 0.57 (0.51, 0.63) | 0.46 (0.39, 0.53) | 0.32 (0.27, 0.37) | 0.32 (0.27, 0.37) |
| Sex of child | Male | 0.59 (0.57, 0.62) | 0.53 (0.50, 0.57) | 0.45 (0.42, 0.48) | 0.42 (0.40, 0.45) |
|  | Female | 0.55 (0.52, 0.58) | 0.50 (0.46, 0.53) | 0.42 (0.39, 0.45) | 0.37 (0.34, 0.40) |
| Age of child in months | 0 - 5 | 0.22 (0.17, 0.26) | 0.17 (0.11, 0.23) | 0.09 (0.06, 0.12) | 0.16 (0.12, 0.20) |
|  | 6 - 23 | 0.50 (0.47, 0.54) | 0.45 (0.41, 0.50) | 0.35 (0.32, 0.39) | 0.34 (0.31, 0.37) |
|  | 24 - 59 | 0.67 (0.64, 0.69) | 0.60 (0.57, 0.63) | 0.53 (0.50, 0.56) | 0.46 (0.44, 0.49) |
| Birth order | First | 0.53 (0.49, 0.57) | 0.48 (0.42, 0.54) | 0.43 (0.39, 0.47) | 0.40 (0.35, 0.44) |
|  | Second | 0.55 (0.52, 0.59) | 0.49 (0.43, 0.55) | 0.41 (0.37, 0.45) | 0.40 (0.36, 0.44) |
|  | Third | 0.58 (0.54, 0.63) | 0.50 (0.44, 0.55) | 0.45 (0.41, 0.49) | 0.37 (0.32, 0.42) |
|  | Forth+ | 0.59 (0.56, 0.62) | 0.54 (0.50, 0.58) | 0.44 (0.41, 0.48) | 0.41 (0.38, 0.43) |
| Water | Improved | 0.57 (0.54, 0.61) | 0.50 (0.47, 0.54) | 0.43 (0.40, 0.46) | 0.40 (0.37, 0.42) |
|  | Unimproved | 0.57 (0.54, 0.60) | 0.53 (0.49, 0.57) | 0.44 (0.41, 0.48) | 0.40 (0.37, 0.42) |
| Sanitation | Improved | 0.63 (0.58, 0.69) | 0.58 (0.49, 0.68) | 0.38 (0.33, 0.43) | 0.40 (0.34, 0.47) |
|  | Unimproved | 0.57 (0.54, 0.59) | 0.51 (0.48, 0.54) | 0.44 (0.42, 0.47) | 0.40 (0.38, 0.42) |
| Handwashing | Improved | n/a | n/a | 0.34 (0.23, 0.46) | 0.39 (0.37, 0.42) |
|  | Unimproved | n/a | n/a | 0.44 (0.42, 0.46) | 0.40 (0.37, 0.43) |
| WASH | Improved | 0.58 (0.49, 0.66) | 0.47 (0.36, 0.58) | 0.20 (0.08, 0.33) | 0.33 (0.25, 0.40) |
|  | Unimproved | 0.57 (0.54, 0.60) | 0.51 (0.48, 0.54) | 0.44 (0.41, 0.47) | 0.40 (0.38, 0.42) |

n/a= not applicable because handwashing variable was not collected; WASH= combined water, sanitation, and handwashing.
