# Supplementary material for: Trends in child growth failure among children under five years of age in Ethiopia: Evidence from the 2000 to 2016 Demographic and Health Surveys
Source: PLoS One. 2021 Aug 5;16(8):e0254768. doi: 10.1371/journal.pone.0254768 (PMC8341490; doi:10.1371/journal.pone.0254768)
Supplement: S2 Table — (DOCX) [file pone.0254768.s003.docx]

**S2 Table. Predicted probabilities for underweight over the four survey years, EDHS.**

| **Variable** | **Category** | **2000** | **2005** | **2011** | **2016** |
| --- | --- | --- | --- | --- | --- |
|  |  | **(95% CI)** | **(95% CI)** | **(95% CI)** | **(95% CI)** |
| Residence | Urban | 0.45 (0.38, 0.52) | 0.28 (0.21, 0.36) | 0.25 (0.17, 0.33) | 0.23 (0.18, 0.27) |
|  | Rural | 0.41 (0.39, 0.44) | 0.34 (0.31, 0.37) | 0.28 (0.26, 0.31) | 0.24 (0.23, 0.26) |
| Region | Tigray | 0.41 (0.37, 0.46) | 0.35 (0.29, 0.42) | 0.34 (0.29, 0.39) | 0.23 (0.20, 0.27) |
|  | Afar | 0.48 (0.41, 0.54) | 0.28 (0.19, 0.37) | 0.38 (0.33, 0.44) | 0.33 (0.27, 0.38) |
|  | Amhara | 0.44 (0.40, 0.48) | 0.44 (0.38, 0.49) | 0.31 (0.27, 0.35) | 0.3 (0.26, 0.33) |
|  | Oromia | 0.39 (0.35, 0.42) | 0.29 (0.25, 0.34) | 0.25 (0.21, 0.29) | 0.22 (0.19, 0.25) |
|  | Somali | 0.41 (0.33, 0.49) | 0.41 (0.34, 0.47) | 0.32 (0.26, 0.37) | 0.25 (0.21, 0.29) |
|  | Beni. Gumuz | 0.38 (0.32, 0.44) | 0.44 (0.36, 0.51) | 0.31 (0.25, 0.37) | 0.34 (0.28, 0.40) |
|  | SNNP | 0.48 (0.43, 0.54) | 0.31 (0.28, 0.35) | 0.27 (0.23, 0.31) | 0.22 (0.19, 0.25) |
|  | Gambela | 0.35 (0.28, 0.42) | 0.24 (0.18, 0.29) | 0.24 (0.18, 0.30) | 0.25 (0.18, 0.31) |
|  | Harari | 0.27 (0.22, 0.33) | 0.28 (0.18, 0.38) | 0.24 (0.19, 0.29) | 0.23 (0.18, 0.27) |
|  | Addis Ababa | 0.20 (0.14, 0.26) | 0.19 (0.11, 0.28) | 0.12 (0.06, 0.18) | 0.12 (0.05, 0.20) |
|  | Dire Dawa | 0.32 (0.25, 0.39) | 0.27 (0.21, 0.34) | 0.29 (0.24, 0.34) | 0.30 (0.24, 0.37) |
| Paternal education | No schooling | 0.43 (0.40, 0.46) | 0.35 (0.32, 0.38) | 0.29 (0.26, 0.32) | 0.26 (0.23, 0.28) |
|  | Primary | 0.41 (0.37, 0.45) | 0.33 (0.29, 0.37) | 0.28 (0.25, 0.31) | 0.22 (0.20, 0.25) |
|  | Secondary | 0.39 (0.33, 0.45) | 0.29 (0.22, 0.35) | 0.19 (0.13, 0.25) | 0.25 (0.18, 0.31) |
|  | Higher | 0.37 (0.24, 0.50) | 0.19 (0.01, 0.38) | 0.19 (0.10, 0.27) | 0.19 (0.12, 0.27) |
| Maternal education | No schooling | 0.43 (0.40, 0.46) | 0.35 (0.32, 0.39) | 0.28 (0.26, 0.31) | 0.25 (0.23, 0.28) |
|  | Primary | 0.39 (0.35, 0.44) | 0.29 (0.24, 0.34) | 0.28 (0.24, 0.31) | 0.21 (0.18, 0.24) |
|  | Secondary | 0.34 (0.24, 0.43) | 0.20 (0.11, 0.29) | 0.16 (0.07, 0.25) | 0.18 (0.12, 0.25) |
|  | Higher | 0.67 (0.36, 0.99) | 0.27 (0.0, 0.54) | 0.10 (-0.0, 0.20) | 0.26 (0.09, 0.43) |
| Maternal age | 15 - 24 | 0.39 (0.35, 0.42) | 0.34 (0.29, 0.38) | 0.29 (0.25, 0.33) | 0.27 (0.24, 0.31) |
|  | 25 - 34 | 0.39 (0.34, 0.44) | 0.36 (0.29, 0.43) | 0.28 (0.22, 0.34) | 0.25 (0.20, 0.31) |
|  | 35 - 44 | 0.46 (0.40, 0.53) | 0.39 (0.30, 0.49) | 0.23 (0.18, 0.28) | 0.24 (0.17, 0.30) |
|  | 45 - 49 | 0.43 (0.40, 0.46) | 0.33 (0.29, 0.36) | 0.28 (0.26, 0.31) | 0.23 (0.21, 0.25) |
| Wealth quintile | Poorest | 0.41 (0.37, 0.45) | 0.35 (0.31, 0.39) | 0.33 (0.29, 0.37) | 0.29 (0.25, 0.33) |
|  | Poorer | 0.42 (0.38, 0.47) | 0.38 (0.33, 0.44) | 0.30 (0.26, 0.34) | 0.28 (0.24, 0.31) |
|  | Middle | 0.44 (0.40, 0.48) | 0.33 (0.28, 0.38) | 0.27 (0.24, 0.31) | 0.23 (0.20, 0.26) |
|  | Richer | 0.40 (0.36, 0.44) | 0.30 (0.25, 0.35) | 0.26 (0.21, 0.30) | 0.18 (0.15, 0.21) |
|  | Richest | 0.40 (0.33, 0.46) | 0.28 (0.22, 0.33) | 0.19 (0.14, 0.24) | 0.20 (0.16, 0.25) |
| Sex of child | Male | 0.44 (0.41, 0.47) | 0.35 (0.31, 0.38) | 0.30 (0.27, 0.33) | 0.25 (0.23, 0.27) |
|  | Female | 0.39 (0.36, 0.42) | 0.33 (0.29, 0.36) | 0.26 (0.23, 0.28) | 0.23 (0.21, 0.25) |
| Age of child in months | 0 - 5 | 0.15 (0.11, 0.19) | 0.12 (0.07, 0.17) | 0.10 (0.07, 0.14) | 0.11 (0.08, 0.15) |
|  | 6 - 23 | 0.42 (0.38, 0.46) | 0.32 (0.28, 0.36) | 0.25 (0.22, 0.29) | 0.22 (0.19, 0.24) |
|  | 24 - 59 | 0.46 (0.43, 0.49) | 0.38 (0.35, 0.41) | 0.32 (0.29, 0.35) | 0.27 (0.25, 0.29) |
| Birth order | First | 0.35 (0.31, 0.39) | 0.30 (0.25, 0.35) | 0.28 (0.24, 0.31) | 0.24 (0.21, 0.28) |
|  | Second | 0.38 (0.34, 0.42) | 0.30 (0.25, 0.35) | 0.25 (0.21, 0.29) | 0.24 (0.20, 0.27) |
|  | Third | 0.43 (0.38, 0.47) | 0.35 (0.30, 0.41) | 0.31 (0.27, 0.35) | 0.23 (0.19, 0.27) |
|  | Forth+ | 0.45 (0.42, 0.48) | 0.36 (0.32, 0.40) | 0.28 (0.25, 0.31) | 0.25 (0.23, 0.27) |
| Water | Improved | 0.42 (0.38, 0.45) | 0.34 (0.30, 0.37) | 0.28 (0.25, 0.31) | 0.25 (0.22, 0.27) |
|  | Unimproved | 0.42 (0.38, 0.45) | 0.34 (0.30, 0.38) | 0.28 (0.25, 0.31) | 0.24 (0.21, 0.26) |
| Sanitation | Improved | 0.50 (0.43, 0.57) | 0.38 (0.27, 0.49) | 0.22 (0.18, 0.27) | 0.24 (0.17, 0.30) |
|  | Unimproved | 0.41 (0.38, 0.44) | 0.33 (0.31, 0.36) | 0.29 (0.26, 0.31) | 0.24 (0.23, 0.26) |
| Handwashing | Improved | n/a | n/a | 0.2 (0.09, 0.32) | 0.23 (0.21, 0.26) |
|  | Unimproved | n/a | n/a | 0.29 (0.27, 0.31) | 0.25 (0.23, 0.28) |
| WASH | Improved | 0.45 (0.36, 0.55) | 0.37 (0.25, 0.49) | 0.18 (0.04, 0.31) | 0.25 (0.16, 0.33) |
|  | Unimproved | 0.41 (0.39, 0.44) | 0.33 (0.31, 0.36) | 0.28 (0.26, 0.31) | 0.24 (0.23, 0.26) |

n/a= not applicable because handwashing variable was not collected; WASH= combined water, sanitation, and handwashing.
