# Supplementary material for: Trends in child growth failure among children under five years of age in Ethiopia: Evidence from the 2000 to 2016 Demographic and Health Surveys
Source: PLoS One. 2021 Aug 5;16(8):e0254768. doi: 10.1371/journal.pone.0254768 (PMC8341490; doi:10.1371/journal.pone.0254768)
Supplement: S3 Table — (DOCX) [file pone.0254768.s004.docx]

**S3 Table. Predicted probabilities for wasting over the four survey years, EDHS.**

| **Variable** | **Category** | **2000** | **2005** | **2011** | **2016** |
| --- | --- | --- | --- | --- | --- |
|  |  | **(95% CI)** | **(95% CI)** | **(95% CI)** | **(95% CI)** |
| Residence | Urban | 0.14 (0.08, 0.20) | 0.13 (0.07, 0.18) | 0.08 (0.05, 0.11) | 0.12 (0.09, 0.16) |
|  | Rural | 0.12 (0.11, 0.14) | 0.11 (0.10, 0.13) | 0.10 (0.09, 0.12) | 0.10 (0.08, 0.11) |
| Region | Tigray | 0.12 (0.09, 0.14) | 0.14 (0.09, 0.18) | 0.11 (0.09, 0.14) | 0.11 (0.08, 0.13) |
|  | Afar | 0.17 (0.13, 0.21) | 0.09 (0.06, 0.12) | 0.21 (0.18, 0.25) | 0.16 (0.12, 0.20) |
|  | Amhara | 0.11 (0.09, 0.14) | 0.16 (0.12, 0.20) | 0.10 (0.08, 0.12) | 0.10 (0.08, 0.12) |
|  | Oromia | 0.11 (0.09, 0.14) | 0.09 (0.06, 0.12) | 0.10 (0.08, 0.12) | 0.10 (0.08, 0.12) |
|  | Somali | 0.18 (0.13, 0.24) | 0.22 (0.16, 0.29) | 0.22 (0.16, 0.27) | 0.21 (0.16, 0.26) |
|  | Beni. Gumuz | 0.16 (0.11, 0.21) | 0.18 (0.11, 0.25) | 0.10 (0.07, 0.13) | 0.10 (0.07, 0.13) |
|  | SNNP | 0.15 (0.12, 0.18) | 0.09 (0.07, 0.12) | 0.08 (0.06, 0.10) | 0.06 (0.04, 0.08) |
|  | Gambela | 0.21 (0.14, 0.27) | 0.10 (0.04, 0.16) | 0.14 (0.09, 0.19) | 0.15 (0.11, 0.20) |
|  | Harari | 0.08 (0.04, 0.12) | 0.13 (0.07, 0.18) | 0.12 (0.08, 0.16) | 0.11 (0.07, 0.16) |
|  | Addis Ababa | 0.08 (0.03, 0.13) | 0.06 (0.0, 0.11) | 0.08 (0.03, 0.12) | 0.06 (0.02, 0.09) |
|  | Dire Dawa | 0.13 (0.09, 0.18) | 0.16 (0.09, 0.23) | 0.14 (0.10, 0.18) | 0.11 (0.07, 0.15) |
| Paternal education | No schooling | 0.13 (0.11, 0.15) | 0.12 (0.10, 0.14) | 0.11 (0.09, 0.13) | 0.10 (0.08, 0.11) |
|  | Primary | 0.12 (0.09, 0.14) | 0.11 (0.08, 0.13) | 0.10 (0.08, 0.11) | 0.09 (0.08, 0.11) |
|  | Secondary | 0.10 (0.06, 0.14) | 0.10 (0.06, 0.14) | 0.05 (0.02, 0.08) | 0.11 (0.07, 0.15) |
|  | Higher | 0.15 (0.02, 0.28) | 0.13 (-0.01, 0.27) | 0.05 (0.02, 0.09) | 0.10 (0.05, 0.14) |
| Maternal education | No schooling | 0.13 (0.11, 0.14) | 0.12 (0.1, 0.14) | 0.11 (0.09, 0.13) | 0.10 (0.08, 0.11) |
|  | Primary | 0.12 (0.09, 0.16) | 0.1 (0.07, 0.14) | 0.08 (0.07, 0.10) | 0.09 (0.07, 0.11) |
|  | Secondary | 0.14 (0.07, 0.20) | 0.05 (0.01, 0.10) | 0.03 (0.0, 0.05) | 0.10 (0.05, 0.15) |
|  | Higher | 0.03 (-0.0, 0.09) | 0.21 (-0.1, 0.54) | 0.10 (0.01, 0.19) | 0.13 (0.03, 0.24) |
| Maternal age | 15 - 24 | 0.12 (0.10, 0.15) | 0.14 (0.10, 0.17) | 0.11 (0.09, 0.14) | 0.11 (0.08, 0.13) |
|  | 25 - 34 | 0.14 (0.10, 0.17) | 0.08 (0.05, 0.12) | 0.11 (0.07, 0.14) | 0.10 (0.07, 0.13) |
|  | 35 - 44 | 0.12 (0.08, 0.17) | 0.14 (0.07, 0.21) | 0.08 (0.05, 0.12) | 0.06 (0.02, 0.11) |
|  | 45 - 49 | 0.12 (0.10, 0.14) | 0.11 (0.09, 0.13) | 0.10 (0.08, 0.11) | 0.10 (0.08, 0.11) |
| Wealth quintile | Poorest | 0.13 (0.10, 0.15) | 0.12 (0.09, 0.14) | 0.12 (0.10, 0.15) | 0.12 (0.10, 0.15) |
|  | Poorer | 0.12 (0.10, 0.15) | 0.15 (0.12, 0.19) | 0.12 (0.10, 0.15) | 0.10 (0.08, 0.12) |
|  | Middle | 0.13 (0.10, 0.15) | 0.12 (0.09, 0.15) | 0.10 (0.08, 0.12) | 0.10 (0.07, 0.12) |
|  | Richer | 0.12 (0.10, 0.15) | 0.08 (0.05, 0.11) | 0.08 (0.06, 0.11) | 0.07 (0.05, 0.10) |
|  | Richest | 0.11 (0.08, 0.15) | 0.08 (0.05, 0.11) | 0.05 (0.03, 0.07) | 0.08 (0.06, 0.11) |
| Sex of child | Male | 0.14 (0.12, 0.16) | 0.12 (0.10, 0.15) | 0.12 (0.10, 0.14) | 0.10 (0.09, 0.12) |
|  | Female | 0.11 (0.09, 0.13) | 0.10 (0.08, 0.12) | 0.08 (0.07, 0.10) | 0.09 (0.08, 0.11) |
| Age of child in months | 0 - 5 | 0.13 (0.10, 0.16) | 0.16 (0.10, 0.21) | 0.15 (0.11, 0.19) | 0.16 (0.12, 0.20) |
|  | 6 - 23 | 0.20 (0.17, 0.23) | 0.15 (0.11, 0.18) | 0.15 (0.12, 0.18) | 0.12 (0.10, 0.15) |
|  | 24 - 59 | 0.08 (0.07, 0.10) | 0.09 (0.07, 0.11) | 0.07 (0.06, 0.09) | 0.07 (0.06, 0.09) |
| Birth order | First | 0.1 (0.08, 0.13) | 0.10 (0.07, 0.14) | 0.09 (0.07, 0.12) | 0.09 (0.07, 0.11) |
|  | Second | 0.11 (0.09, 0.14) | 0.12 (0.09, 0.15) | 0.10 (0.07, 0.12) | 0.06 (0.05, 0.08) |
|  | Third | 0.12 (0.10, 0.15) | 0.11 (0.08, 0.14) | 0.10 (0.07, 0.12) | 0.11 (0.08, 0.14) |
|  | Forth+ | 0.14 (0.12, 0.16) | 0.12 (0.09, 0.14) | 0.11 (0.09, 0.13) | 0.11 (0.09, 0.13) |
| Water | Improved | 0.13 (0.11, 0.15) | 0.12 (0.10, 0.15) | 0.11 (0.09, 0.13) | 0.10 (0.08, 0.11) |
|  | Unimproved | 0.12 (0.10, 0.14) | 0.10 (0.08, 0.13) | 0.09 (0.08, 0.11) | 0.10 (0.08, 0.11) |
| Sanitation | Improved | 0.14 (0.08, 0.20) | 0.10 (0.05, 0.15) | 0.08 (0.05, 0.10) | 0.10 (0.07, 0.14) |
|  | Unimproved | 0.12 (0.11, 0.14) | 0.12 (0.10, 0.13) | 0.10 (0.09, 0.12) | 0.10 (0.09, 0.11) |
| Handwashing | Improved | n/a | n/a | 0.06 (-0.02, 0.13) | 0.10 (0.08, 0.12) |
|  | Unimproved | n/a | n/a | 0.10 (0.09, 0.11) | 0.09 (0.08, 0.11) |
| WASH | Improved | 0.15 (0.06, 0.23) | 0.14 (0.06, 0.21) | 0.04 (-0.0, 0.09) | 0.12 (0.06, 0.18) |
|  | Unimproved | 0.12 (0.11, 0.14) | 0.11 (0.10, 0.13) | 0.10 (0.08, 0.12) | 0.10 (0.09, 0.11) |

n/a= not applicable because handwashing variable was not collected; WASH= combined water, sanitation, and handwashing.
